# Effective Removal of Cd(II) from Aqueous Solutions Using *Theobroma cacao* Agro-Industrial Waste

**DOI:** 10.3390/molecules28145491

**Published:** 2023-07-18

**Authors:** Carmencita Lavado-Meza, Leonel De la Cruz-Cerrón, Carmen Lavado-Puente, Francisco Gamarra-Gómez, Elisban Sacari-Sacari, Juan Z. Dávalos-Prado

**Affiliations:** 1 Escuela Profesional de Ingeniería Ambiental, Universidad Nacional Intercultural de la Selva Central Juan Santos Atahualpa, Chanchamayo 12856, Peru; 2 Facultad de Ingeniería, Universidad Continental, Huancayo 12000, Peru; 3 Laboratorio de Nanotecnología, Facultad de Ingeniería, Universidad Nacional Jorge Basadre Grohmann, Av. Miraflores s/n, Tacna 23003, Peru; 4 Instituto de Química Física “Blas Cabrera”, CSIC, 28006 Madrid, Spain

**Keywords:** agro-industrial waste, *Theobroma cocoa*, Cd(II) removal, binary and ternary systems, Pb(II) and Cu(II) co-cations

## Abstract

*Theobroma cacao* agro-industrial waste (WTC) has been characterized and tested as an effective biosorbent to remove Cd(II) from aqueous media. At the optimum pH of 5.0, a maximum adsorption capacity of q_e,max_ = 58.5 mg g^−1^ was determined. The structural and morphological characterization have been conducted by FTIR, SEM/EDX, and TGA measurements. The SEM/EDX results confirmed that the metals are adsorbed on the surface. C-O-C, OH, CH, NH, and C=O functional groups were identified by FTIR. TGA results were consistent with the presence of hemicellulose. Biosorption kinetics were rapid during the first 30 min and then reached equilibrium. The corresponding experimental data were well fitted to pseudo-first and -second order models, the latter being the best. The biosorption isotherm data were also well fitted to Temkin, Langmuir, and Freundlich models, showing that several sorption mechanisms may be involved in the Cd(II) biosorption process, which was characterized as exothermic (ΔH^0^ < 0), feasible, and spontaneous (ΔG^0^ < 0). In binary (Cd–Pb and Cd–Cu) and ternary (Cd–Pb–Cu) systems, Cu(II) and particularly Pb(II) co-cations exert strong antagonistic effects. Using HNO_3_, effective good regeneration of WTC was obtained to efficiently remove Cd(II) up to three times.

## 1. Introduction

Nowadays, many drinking water and agricultural irrigation sources are contaminated by heavy metals, representing a threat to human health even when at minimal concentrations within the body [1,2]. Cadmium (Cd) is an extremely dangerous heavy metal for people, animals, and plants [3]. It readily accumulates in the food chain to reach humans, causing severe damage to the renal, reproductive, nervous, hepatic, or pulmonary systems; it also affects growth, bone metabolism, and can even cause cancer [4]. Therefore, the EPA (USA-Environmental Protection) has included cadmium in the list of priority pollutants and the WHO (World Health Organization) has set the maximum permissible concentration at 0.003 mg L^−1^ in drinking water and industrial wastewater [5].

The environmental presence of Cd comes from various sources, particularly industrial ones related to the production and recycling of batteries [6,7], alloys, coatings (galvanoplasty), solar cells, plastic stabilizers, or pigments. Therefore, Cd removal is a very current topic of interest. There are traditional methods to remove cadmium from contaminated aqueous solutions: filtration, ion exchange, chemical oxidation or reduction, chemical precipitation, evaporative recovery, and electrochemical treatment [3,8]. However, these methods are not very effective for contaminated aqueous solutions with Cd concentrations below 100 mg L^−1^ [9]. In this context, the use of specific biomass becomes an effective, economical, and easy-to-operate alternative [10]. Biosorption is defined as the ability of biomass (non-living and inactive biological matter) to retain toxic metals from aqueous media through the physicochemical processes occurring between metal ions and the biomass binding sites. Biosorption involves processes such as complex formation, electrostatic interactions, ion exchange, precipitation, etc. [11,12]. The literature reports information on different biosorbents from agricultural waste for Cd(II) removal, such as *Phyllanthus emblica* fruit stone [13], rice husk [5], avocado pear exocarp [14], olive leaves [15], and moringa oleifera leaves [16]. However, the information on the use of cocoa waste for the removal of this metal is rather scarce. It is important to highlight that Peru is one of the largest cocoa producers in the world, with a production of 218 KTn year^−1^ that generates approximately 153 KTn year^—1^ of waste. Thus, cocoa residues could be a sustainable resource for the production of heavy metal biosorbents [17,18,19,20] with potential applications at an industrial level, within a circular economy that uses eco-friendly materials with a low environmental impact. In this context, one of our objectives is the search for biomass, capable of efficiently removing heavy metals at a low economic cost and with the guarantee of a sustained supply. In the present study, we evaluated the capacity of *Theobroma cacao* agro-industrial waste (WTC) as an efficient and economic biomass to remove Cd(II) from aqueous solutions, for which we studied the influence of pH, dosage, Cd(II) initial concentration and the effect of Cu(II) and Pb(II) as co-cations in binary and ternary metal removal systems.

## 2. Results and Discussion

### 2.1. Biosorbent Characterization

**Figure 1:**
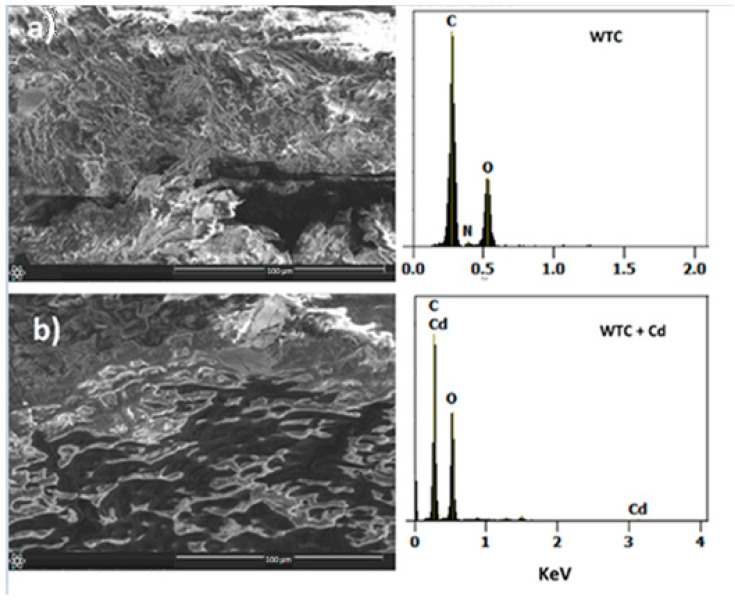
SEM (left) and EDX (right) of WTC, (**a**) before and (**b**) after Cd(II) biosorption. pH 5.0, C_o_ = 100 mg L^−1^, *T* = 20 °C, t = 120 min, biomass dosage = 1.0 g L^−1^.

Figure 1a shows the morphology of the clean WTC surface, where it can be observed to have a rough, irregular, and porous surface with a heterogeneous appearance. The observed porosity would favor the number of adsorption sites [17]. The morphology of the surface after Cd(II) adsorption is very different to the clean surface: it is less rough and heterogeneous (Figure 1b). The EDX analysis of clean WTC shows C, N, and O peaks related to its lignocellulosic nature, while WTC loaded with Cd(II) (WTC + Cd) shows Cd peaks in addition to those mentioned, which is evidence of Cd adsorption on the WTC surface.

**Figure 2:**
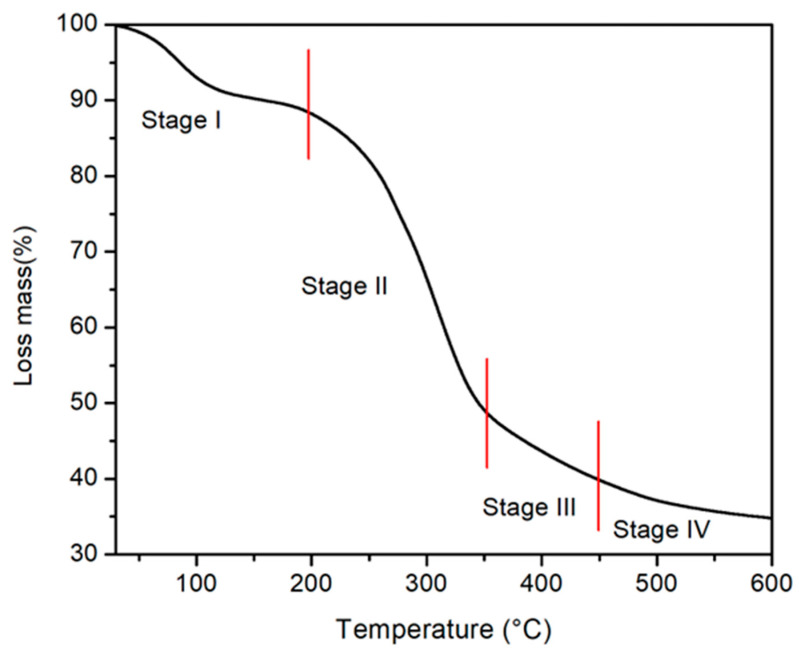
TGA profiles of WTC.

The thermogravimetric (TGA) analysis (Figure 2) shows the thermal degradation of WTC with increasing temperature. We can see different stages of mass loss over temperature: (a) Stage I (from room temperature to 200 °C), the mass loss of ~10% could be attributed to the evaporation of moisture present in the sample [21]. (b) Stage II (200 to 357 °C), the loss of mass is abrupt, up to ~44%, which is related to the degradation of the hemicellulose. (c) Stage III (357 to 450 °C), the mass loss is of the order of 10% and is related to the pyrolysis of cellulose. (d) Stage IV (450 to 600 °C) the loss of mass is slower and would be related to the decomposition of lignin. Therefore, TGA analysis shows that hemicellulose, which is the main component of WTC, degrades more easily than cellulose, and this, in turn, degrades more easily than lignin. These results are similar to those obtained by Basu et al. (2017) [22] with cucumber peel biomass.

**Figure 3:**
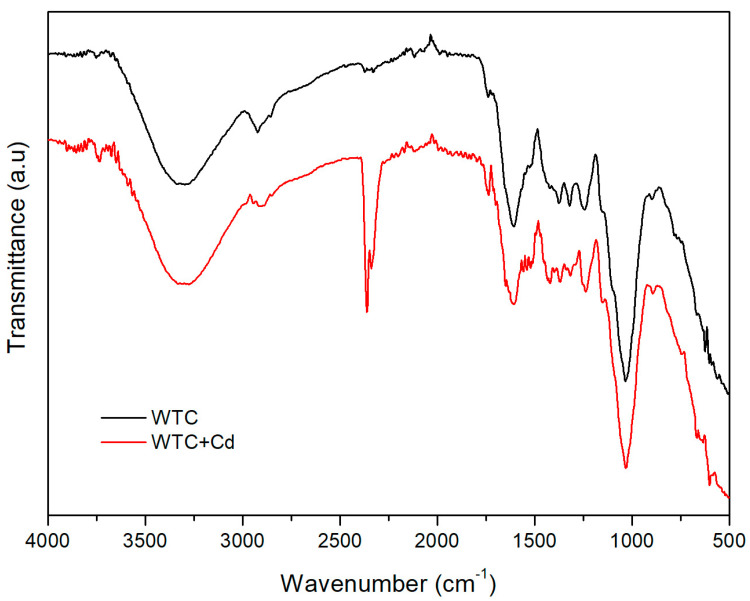
FTIR spectrum of WTC before and after Cd(II) biosorption. C_o_ = 100 mg L^−1^, t = 120 min, dose = 1.0 g L^−1^, *T* = 20 °C, pH 5.0.

Figure 3 displays FT-IR spectra of both clean (WTC) and Cd(II)-loaded WTC (WTC + Cd). For WTC, we observe absorption bands: (a) wide between 3370.4–3236.1 cm^−1^, associated with O-H and N-H stretching [23]; (b) at 2920 cm^−1^, assigned to C-H stretching typical in polysaccharides [24]; (c) around 1736.8 cm^−1^, assigned to C=O stretching of carboxylic groups [25], (d) at 1233 cm^−1^, characteristic of C=O stretching in tertiary amides; (e) at 1031.3 cm^−1^, associated to C-O-C stretching, typical in polysaccharides [26]; (f) at 2330 and 2359 cm^−1^, which can be assigned to O=C=O stretching [27], although we do not rule out the presence of nitrile C≡N groups [28,29]. After biosorption, the (WTC + Cd) spectrum shows changes in the position (red-shifting) of some important bands. Therefore, the bands associated with (C-O-C) and C=O moved, respectively, to 1041.2 and 1242.8 cm^−1^. These changes reflect the interaction of Cd(II) cations with the active sites of WTC [30].

### 2.2. pH Effects

**Figure 4:**
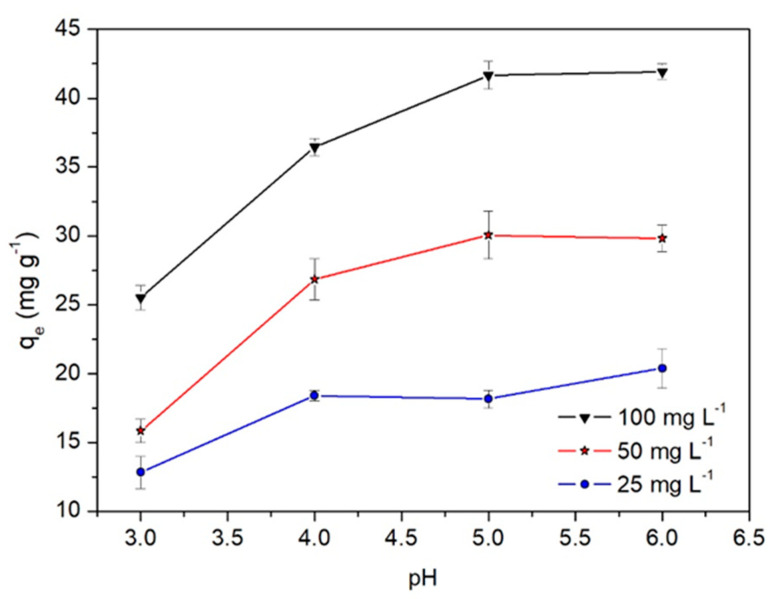
pH effect on the Cd(II) adsorption capacity q_e_. t = 120 min, dose = 1.0 g L^−1^, T = 20 °C.

The initial pH of the solution is one of the important factors for the biosorption process since it affects the surface charge of the biosorbent and the ionization of the metallic sorbate [31]. Figure 4 displays the effect of the initial pH on the Cd(II) adsorption capacity, q_e_, at different initial concentrations of Cd(II), C_o_. For all C_o_ concentrations, a rapid increase in q_e_ is observed in the very acidic pH range (pH ≤ 4). In the pH range from 4 to 5, the increase in q_e_ slows down until reaching a plateau at pH 6 for high C_o_ concentrations. Thus, pH 5.0 has been chosen as the most adequate for the optimal Cd(II) biosorption. These results can be interpreted through the electrostatic interaction mechanism between Cd(II) and the charged WTC surface since the point zero-charge pH of WTC is pH_PZC_ = 3.9 [18]. This indicates that for pH < pH_PZC_ = 3.9, the WTC surface is positively charged and the repulsion with Cd(II) cations limits its adsorption capacity. In contrast, for pH ≥ pH_PZC_, the WTC surface is negatively charged, facilitating Cd(II) adsorption. In addition, under these conditions, the deprotonation of carboxylic (-COOH) and hydroxyl functional groups (identified by FTIR) would occur, for which the pKa is between 4 and 6.

### 2.3. Biosorbent Dosage Effect

**Figure 5:**
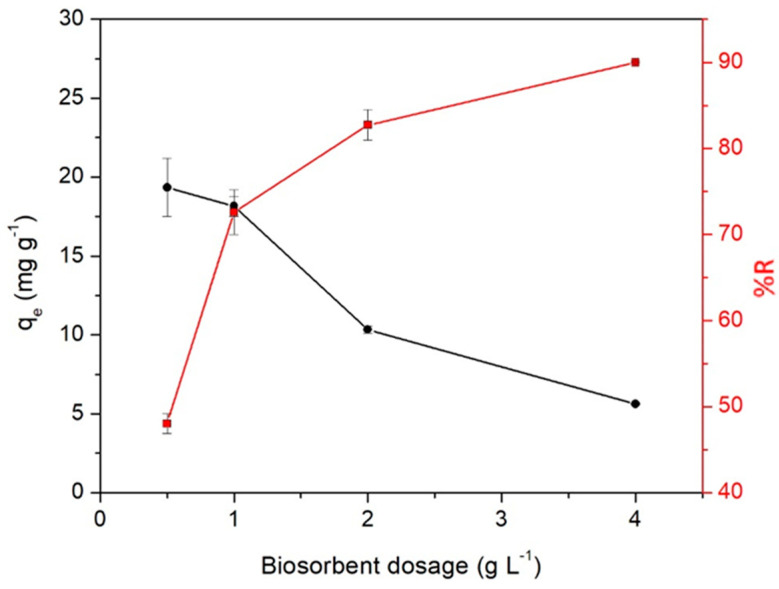
WTC dosage effect on the adsorption capacity (q_e_) and removal efficiency (%R) of Cd(II) at pH 5.0 and C_o_ = 25 mg L^−1^.

Figure 5 displays the dosage effect of WTC on both the adsorption capacity (q_e_) and removal efficiency (%R) of Cd(II). %R increases with an increasing dose of WTC from 0.5 to 4.0 g L^−1^. In contrast, q_e_ decreases in this dose range. At 1.0 g L^−1^, %R = 72% and it increased to maximum removal value (90%) at 4.0 g L^−1^. This would be due to an increase in the surface area with a large number of biosorption vacant sites. For low values of the dose (range 0.5 to 1.0 g L^−1^), all adsorbent sites would be fully occupied and the surface would be saturated, resulting in high q_e_ (~18 mg g^−1^) values [32]. The significant decrease in q_e_ at doses ≥ 1.0 g L^−1^ would be due to the splitting effect of the concentration gradient between the biosorbent and the sorbate with increasing WTC dose, which would cause a reduction in the amount of Cd(II) adsorbed per unit mass of WTC [33,34]. A considerable increase in the biosorbent dosage could also cause its agglomeration and therefore a blockage of the available adsorption sites, reducing the interaction of the metal with the surface [15]. Taking into account these results, a WTC dosage of 1 g L^−1^ was selected as the optimum value that provides an adequate surface area for Cd(II) adsorption.

### 2.4. Kinetic Data

**Figure 6:**
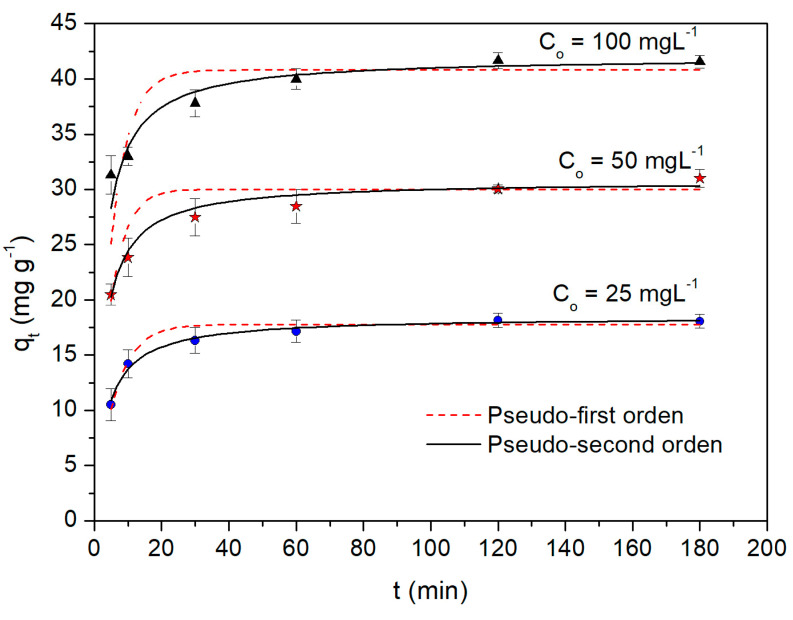
Biosorption kinetics of Cd(II) onto WTC (q_t_ vs. t), at different initial concentrations of Cd(II), C_o_. *T* = 20 °C, dose = 1g L^−1^, pH 5.0.

Figure 6 displays the Cd(II) biosorption capacity, q_t_, as a function of the contact time t with the WTC biosorbent (q_t_ vs. t), for different initial concentrations of the Cd(II) cation, C_o_. For all cases, we can appreciate the rapid increase in q_t_ during the first 30 min. Thereafter, the growth of q_t_ slows to reach equilibrium near t = 120 min. The experimental data obtained were fitted to the pseudo-first and psuedo-second order kinetic models. The corresponding optimized parameters are presented in Table 1. The best fit was obtained with the pseudo-second order model (R^2^ ≥ 0.93, χ^2^ ≤ 0.17), indicating that the Cd(II) biosorption onto WTC would be mainly a chemisorption process [35]. However, the pseudo-first order model also presents a good fit, particularly at low C_o_ concentrations, (R^2^ ≥ 0.89, χ^2^ ≤ 1.19), which shows that in the Cd(II) biosorption process, physical adsorption also takes place, with adsorption rate constants k_1_, of almost 0.2 min^−1^, very similar to that obtained by Tejada-Tovar et al. (2020) [36], in the Pb(II) biosorption onto sugarcane bagasse.

**Figure 7:**
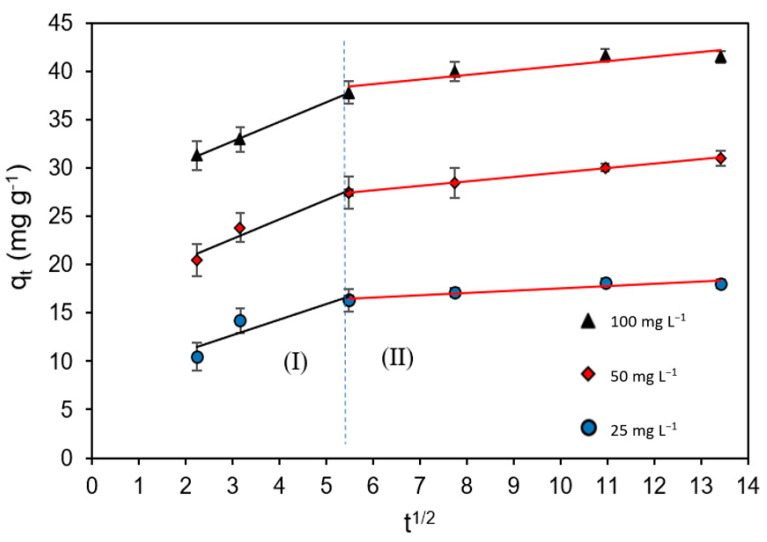
Weber–Morris plots of Cd(II) biosorption onto WTC.

In many cases, there is the possibility that intraparticle diffusion is the rate-limiting step of adsorption, which is usually determined using the equation proposed by Weber and Morris (intraparticle diffusion model) [37] (Table 1). In the q_t_ vs. t^1/2^ plots (Figure 7) we can distinguish two well-defined parts. The first part, 0 < t^1/2^ < 5.41, shows a rapid growth of q_t_ (t), which would indicate a rapid adsorption of Cd(II) cations onto WTC surface. The lines of fit do not pass through the origin of coordinates, which indicates that intraparticle diffusion would not be the only process that controls the rate of adsorption, but that other processes can also control this rate. In the second part, t^1/2^ > 5.41, the slower growth of q_t_ (t) would be related to a gradual adsorption process, where Cd(II) cations would enter and fill the biosorbent pores until the equilibrium is reached [14].

### 2.5. Biosorption Isotherms

**Figure 8:**
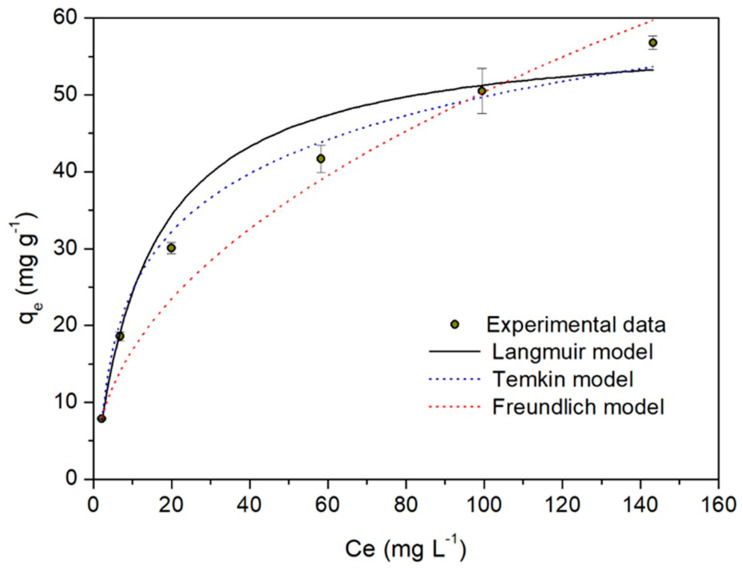
q_e_ vs. C_e_ biosorption isotherms. WTC dose = 1.0 g L^−1^, *T* = 20 °C, t = 120 min, pH 5.0.

The adsorption isotherm provides information about the affinity that a sorbent displays for a particular sorbate [22]. Figure 8 shows the Cd(II) biosorption isotherm onto WTC. The experimental data obtained were adjusted (non-linear adjustments) to three models, Temkin, Langmuir, and Freundlich. The corresponding optimized parameters are consigned in Table 2. With these three models, reasonably acceptable adjustments (R^2^ ≥ 0.96 and χ^2^ ≤ 3.42) have been obtained, although the best adjustment was obtained with the Temkin model.

Thus, the Temkin parameters, particularly that related to heat adsorption, b_T_ = 0.22 kJ mol^−1^ (<8 kJ mol^−1^) indicate that the Cd(II) biosorption onto WTC would be predominantly a physisorption process [38,39,40] where the Cd(II) adsorbates adhere to the WTC adsorbent through weak van der Waals interactions.

The Langmuir model suggests that Cd(II) biosorption onto WTC occurs with the formation of a monolayer, with a maximum adsorption capacity, q_max_ = 58.5 mg g^−1^ and an affinity constant K_L_ = 0.068 L mg^—1^; which, due to its small value, indicates a typical sorbate–sorbent interaction in physisorption processes [40].

The Freundlich model parameters, K_F_ = 5.63 mg^0.53^ g^−1^ L^0.474^ and particularly *n* = 2.11 (*n* > 1), indicate the favorable biosorption of Cd(II) onto WTC.

The kinetic and isotherm adsorption results show that the Cd(II) biosorption process onto WTC would occur through various adsorption mechanisms, such as physisorption, chemisorption, and intraparticle diffusion.

The maximum Cd(II) biosorption capacities, q_e,max_ of biomasses similar to that studied in this work are shown in Table 3. We can appreciate that the q_e,max_ of WTC is among the highest values, indicating that this material is viable for the aqueous removal of Cd(II).

**Table 3:** Maximum Cd(II) adsorption capacities, q_e,max_, of biosorbents coming from agro-industrial waste.

Adsorbent	dose (g L^−1^)	q_e,max_ (mg g^−1^)	References
*Foeniculum vulgari* biomass	10	26.59	[41]
Cocoa pod waste	10	12.15	[42]
Dried cactus (*Opuntia ficus indica*)	0.5	30.42	[43]
Cabbage waste Canola biomass	5 3	20.6 25.86	[44] [45]
Barley husk biomass	3	24	[46]
Peanut husk	3	29	[47]
*Cassia fistula* biomass	2.5	7.24	[48]
Oak waste materials	5	155.9	[49]
Avocado pear exocarp	0.5	13.91	[14]
Spent coffee grounds	2.5	11	[37]
*Agave angustifolia* biomass	6.7	34.84	[50]
*Opuntia fuliginosa* biomass	6.7	30.21	[50]
*Phyllanthus emblica* fruit stone	1	3.15	[13]
Cocoa (*Theobroma cacao*) pod husk	20	4.42	[51]
*Chrysopogon zizanioides* root powder	1	26.69	[30]
*Theobroma cacao* agro-industrial waste	1	58.5	This work

### 2.6. Biosorption Thermodynamics

Thermodynamic functions, such as standard Gibbs energy (Δ*G*^o^), enthalpy (Δ*H*^o^), and entropy changes (Δ*S*^o^) for the Cd(II) biosorption process were evaluated using Equation (1) and the Van’t Hoff Equation (2). All these values are presented in Table 4.

(1) $$\Delta G^{o}=-RT\ln\left(K_{c}\right)$$

(2) $$ln\;K_{c}=\frac{{\Delta S}^{o}}{R}-\frac{{\Delta H}^{o}}{RT}$$

where K_c_ is the equilibrium constant, $K_{c}=\frac{C_{es}}{C_{e}}$, and C_es_ and C_e_ are the equilibrium Cd(II) concentrations, respectively, in the biosorbent and in the solution, *R* is the universal gas constant, and *T* is the temperature of the solution.

Negative values of Δ*G*^o^ and Δ*H*^o^ indicate that the biosorption process is spontaneous and exothermic. Since Δ*H*^o^ values can provide information on characteristic binding energies such as electrostatic (6–80 kJ mol^−1^) or hydrogen bonds (4–13 kJ mol^−1^) [52]; the Δ*H*^o^ value obtained in this work (−8.92 kJ mol^−1^) indicates that electrostatic interactions and hydrogen bonds would be present in the biosorption of Cd(II) onto WTC, which is in good agreement with the kinetics and adsorption isotherm results.

It can also be observed that Δ*G*^o^ values increase or are “less spontaneous” with increasing temperature, showing that the biosorption process is less favorable at high temperatures. In contrast, the negative value of Δ*S*^0^ confirms the decrease in randomness at the solid–liquid interface during biosorption [53].

### 2.7. Effect of Co-Cations in Binary and Ternary Systems

Wastewater usually contains more than one metal (multimetallic aqueous solutions) and the removal by adsorption of each of them is affected by the presence of the other co-cations, which can produce up to three types of behavior: synergism, antagonism, or non-interaction [54]. Antagonistic effects are usually reported in the adsorption capacities of multimetallic solutions, which would be caused by competition among co-cations for the binding sites of the biomass [55].

**Figure 9:**
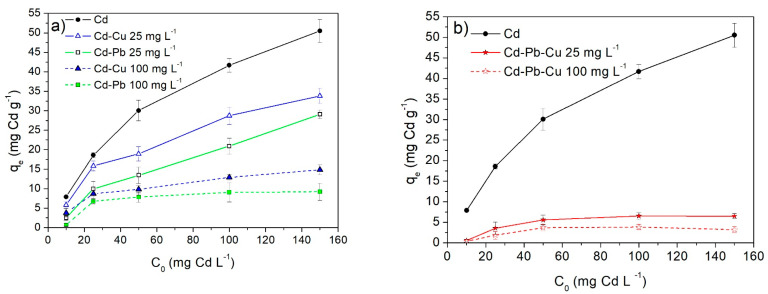
Pb(II) and Cu(II) co-cation effects on the Cd(II) biosorption capacity, q_e_,_Cd_. Initial concentrations of Pb(II) and Cu(II), C_o_ = 25 and 100 mg L^−1^. (**a**) Binary and (**b**) ternary systems.

Figure 9 displays the influence of the initial concentration of Pb(II) and Cu(II) co-cations on the Cd(II) biosorption capacity onto the WTC. As can be seen, the antagonistic effect of co-cations is significant, particularly of Pb(II). Therefore, (a) in the binary systems (Cd-Cu) and (Cd-Pb) (Figure 9a), for low concentrations of co-cations (C_o_ = 25 mg g^−1^), the presence of Cu(II) and Pb(II) reduce q_e_ by up to 33% and 42%, respectively. For high co-cation concentrations (C_o_ = 100 mg g^−1^), the q_e_ reductions can reach up to 71% (Cu) and 82% (Pb). (b) In the ternary system (Figure 9b), the reduction of q_e_ is even more drastic, reaching more than 90%. Similar behavior was reported by Hossain et al. 2014 [44] in a ternary (Cd-Cu-Pb) sorption system of the agro-waste cabbage biosorbent.

Although the mechanisms of multimetallic removal are not sufficiently clarified, the competition between ionic sorbates, such as Pb(II), Cd(II), or Cu(II), for occupying the adsorption sites, is evident. Basu et al. (2017) [22], among others, highlight the importance of metallic sorbate parameters such as electronegativity and hydrated ionic radius. It is known that Pb and Cu have high electronegativity values (1.8 and 1.9, respectively) [56], but a low hydrated ionic radius (410 and 419 pm) [57] in comparison with Cd (1.7 and 426 p.m.). As they are more electronegative and have smaller hydrated ionic radii, both Pb(II) and Cu(II) would be more competitive cations than Cd(II). Therefore, these co-cations would antagonistically affect the Cd(II) biosorption capacity in binary systems and much more in ternary systems. Furthermore, Hossain et al. 2014 [44] have reported that the most binding sites of biosorbent (cabbage) are occupied by Pb(II) ions in competitive binary, ternary and quaternary systems.

### 2.8. Desorption

The regeneration of a biosorbent, such as WTC, is crucial for biomass reuse, and metal recovery, and also to reduce the operating costs of any type of water treatment [58]. The literature reports a high desorption yield using HNO_3_ as an eluent or desorbing agent [18,59,60]. This acid acts as an effective ion exchange medium, since its released H^+^ replaces the Cd(II) cation on the WTC surface, allowing efficient Cd(II) desorption [35,44].

**Figure 10:**
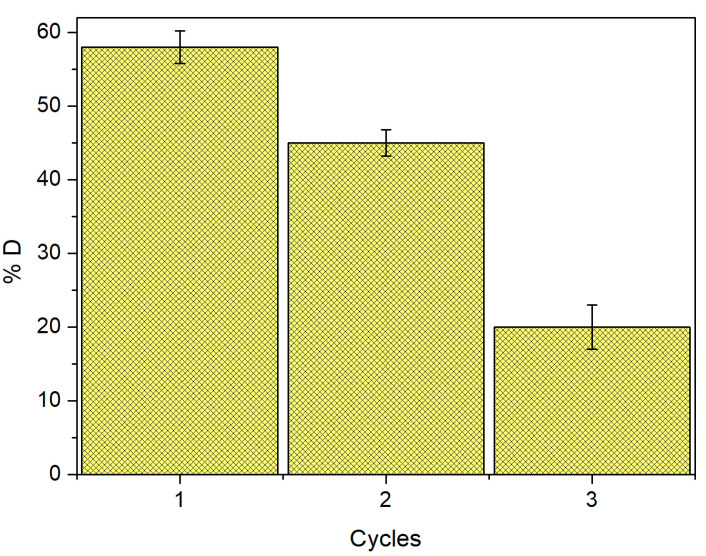
Regeneration efficiency %D of WTC vs. a number of Cd(II) adsorption/desorption cycles. Biomass dose = 1.0 g L^−1^.

Figure 10 shows the desorption capacity of WTC using HNO_3_. In the first cycle of Cd(II) removal, a desorption of %D = 58% is reached; in the second cycle, 45%; and in the third cycle, up to 20%.

## 3. Materials and Methods

### 3.1. Preparation and Characterizations of WTC Biosorbent

The preparation of the biosorbent is quite simple and friendly to the environment. Agro-industrial waste from *Theobroma cacao* was collected from the Chanchamayo province located in the Junin-region of Peru. Samples were first washed with water, rinsed with distilled water, and dried at 70 °C for 48 h. Subsequently, the dried adsorbent was ground and sieved using a 70-mesh. All the chemical products used in this work were of analytical grade and from Sigma-Aldrich (Steinheim, Germany) or Merck, Darmstadt, Germany. The Cd(II) stock solution (1000 mg L^−1^) was prepared from Cd(NO_3_)_2_.4H_2_O. To obtain solutions at different concentrations, appropriate amounts of the stock solution were diluted.

Thermogravimetric analysis (TGA) of WTC was performed using Thermo Scientific spectrometer model Evolution 220 (Thermo Scientific Co., Ltd., Waltham, DE, USA) in an N_2_ atmosphere (100 mL min^−1^). The heating rate of WTC was 10 °C min^−1^ with temperatures ranging from 20 to 600 °C.

The functional groups on the WTC surface were identified using Perkin Elmer Frontier Model Spectrometer (PerkinElmer Inc., Wellesley, MA, USA) equipped with a Platinum ATR accessory. Spectral data were collected over the wavenumber range from 400 to 4000 cm^−1^. The morphological analysis and elemental composition before and after Cd(II) biosorption were obtained using SEM/EDX (Thermo Scientific Co., Eindhoven, The Netherlands).

### 3.2. Biosorption Assays

**Figure 11:**
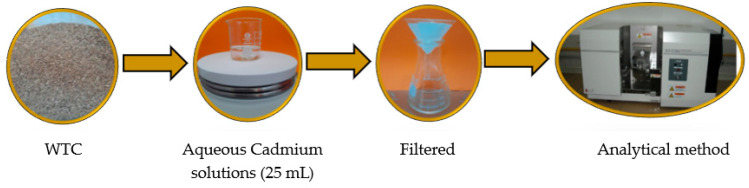
Scheme of cadmium adsorption procedure by WTC.

pH, dose of biomass, and initial Cd(II) concentration effects on the adsorption capacity of this metal were evaluated. At room temperature (20 °C), amounts of WTC biomass between 0.0125 and 0.1 g were mixed with 25 mL of Cd(II) solution with a concentration that varied from 10 to 200 mg L^−1^ (Figure 11). The pH of these solutions was adjusted in the range from 3 to 6 by adding appropriate amounts of 0.1 M HNO_3_ or 0.1 M NaOH. The obtained suspension was stirred at 300 rpm for 120 min at room temperature. The initial concentration before and after Cd(II) adsorption in each experiment was determined by atomic absorption spectroscopy (Shimadzu-AAS 6800, Kyoto, Japan). The adsorption capacity, q_e_, and removal efficiency, %R, were determined by Equations (3) and (4), respectively:

(3) $$q_{e}=\frac{\left(C_{0}-C_{e}\right)\times V}{m}$$

(4) $$\%R=\frac{\left(C_{o}-C_{e}\right)}{C_{0}}\;\times \;100$$

where C_o_ and C_e_ (in mg·L^−1^) are the initial and equilibrium final Cd(II) concentrations, respectively; V (in L) is the volume of solution; and m (in g) is the biosorbent mass.

All experiments were performed in triplicate. The mean and corresponding standard deviation are reported. Both kinetics and biosorption–isotherm experimental data were adjusted according to the different models considered and are described in Table 5. The quality of the adjustments was evaluated with the chi-square χ^2^ and correlation coefficient R^2^ parameters. The adjustment criteria that indicate the suitability of the model were low values for χ^2^ and close to unity for R^2^ [61].

### 3.3. Competitive Effect of Co-Cations

The experiments were carried out in a mixed system, varying the Cd(II) initial concentrations from 10 to 150 mg L^−1^, keeping constant the initial concentration of Pb(II) and/or Cu(II) co-cations (25 and 100 mg L^−1^). A WTC dose of 0.025 g and 25 mL of mixed solution at pH 5.0 were used.

### 3.4. Desorption Experiments

First, 50 mg of WTC, previously loaded with Cd(II) (C_o_ = 100 mg L^−1^), was subjected to the desorption process by adding 50 mL of 0.1 M HNO_3_ eluent and then stirring at 300 rpm for 120 min. Subsequently, the biosorbent was washed with distilled water and reused again. The adsorption/desorption operation was repeated up to three times. The concentration of Cd(II) adsorbed and desorbed was analyzed by an atomic absorption spectrophotometer (described above). The Cd(II) desorption efficiency (%D) of WTC was calculated using the following expression [35]:

(5) $$\%D=\frac{Cd\left(II\right)desorbed}{Cd\left(II\right)sorbed}\times 100$$

## 4. Conclusions

The feasibility and efficiency of *Theobroma cacao* agro-industrial waste (WTC) for Cd(II) biosorption in aqueous solutions has been studied. This eco-friendly and low-cost biomass was characterized by SEM/EDX, TGA, and FT-IR techniques. TGA analysis indicates the presence of hemicellulose in the WTC structure. SEM results show that the WTC morphology changes Cd(II) biosorption. FT-IR spectra show the bands associated with the OH, NH, CH, and C-O-C functional groups typical of polysaccharides; and to C=O carboxylic groups. After Cd(II) adsorption, several of these bands were red-shifted and changed in intensity, revealing the interaction between Cd(II) and the WTC surface. It has been determined optimal biosorption conditions: pH 5.0, WTC dosage = 1.0 g L^−1^, adsorption equilibrium time, 120 min. Kinetic experimental data fit quite well both to the pseudo-second and pseudo-first order models, which implies that physisorption and chemisorption processes would be involved in the Cd(II) biosorption. The biosorption isotherm data fit well with the Temkin, Langmuir, and Freundlich models, which shows that several sorption mechanisms would be involved in the Cd(II) biosorption process onto WTC. From the adjustment to the Langmuir model, the maximum biosorption capacity was derived, q_e,max_ = 58.5 mg g^−1^; this is one of the highest values reported in the literature for similar biomasses coming from agro-industrial waste. The thermodynamic study indicates that the Cd(II) biosorption process is exothermic (Δ*H*^o^ = −8.9 kJmol^−1^), spontaneous (Δ*G*^o^ < 0), and with decreasing randomness (Δ*S*^o^ < 0) at the solid–liquid interface. The presence of Pb(II) and Cu(II) co-cations in binary and particularly in ternary systems significantly reduces the Cd(II) biosorption capacity onto WTC; with Pb(II) being the co-cation that exerts the strongest antagonistic effect. The desorption experiments, using HNO_3_ as the eluent, showed that the recovery of WTC is feasible for reused up to three times.

## Figures and Tables

**Table 1:** Kinetic parameters for Cd(II) biosorption onto WTC. *T* = 20 °C, pH 5.0, dose = 1.0 g L^−1^.

Model	Parameters	C_o_ = 25 mg L^−1^	50 mg L^−1^	100 mg L^−1^
Pseudo-first order	q_e,cal_ (mg g^−1^)	17.78 ± 0.23	30.08 ± 0.43	40.82 ± 0.99
	k_1_ (min^−1^)	0.17 ± 0.021	0.22 ± 0.025	0.19 ± 0.033
	R^2^ χ^2^	0.89 0.16	0.90 1.19	0.86 8.47
Pseudo-second-order	q_e,cal_ (mg g^−1^)	18.47 ± 0.16	30.76 ± 0.23	41.99 ± 0.53
	k_2_ (g mg^−1^·min^−1^)	0.015 ± 0.001	0.012 ± 0.001	0.010 ± 0.001
	h (mg g^−1^·min^−1^)	5.12	12.30	17.63
	R^2^	0.98	0.93	0.98
	χ^2^	0.05	0.17	0.04
Intra-particle diffusion	k_id,I_ (mg g^−1^· min^−1/2^) B_I_ R^2^ χ^2^	1.62 ± 0.02 7.80 ± 0.52 0.90 0.12	2.05 ± 0.03 16.51 ± 0.83 0.95 0.024	2.02 ±0.03 26.69 ± 0.92 0.99 0.015
	k_id,II_ (mg g^−1^· min^−1/2^) B_II_ R^2^ χ^2^	0.23 ± 0.01 15.27 ± 0.78 0.90 0.10	0.45 ± 0.01 25.01 ± 0.81 0.99 0.026	0.47 ± 0.02 35.81 ± 0.94 0.85 0.015

**Table 2:** Cd(II) biosorption isotherm parameters.

Model	Parameters	
Langmuir	q_max_ (mg g^−1^)	58.5 ± 3.6
	K_L_ (L mg^−1^)	0.068 ± 0.007
	R^2^ χ^2^	0.98 2.41
Freundlich	n	2.11 ± 0.02
	K_F_ (mg^0.53^ g^−1^ L^0.47^)	5.63 ± 0.44
	R^2^ χ^2^	0.96 3.42
Temkin	b_T_ (J mol^−1^) K_T_ (Lg^−1^) R^2^ χ^2^	0.22 ± 0.03 0.95 ± 0.04 0.99 1.85

**Table 4:** Thermodynamic parameters of Cd(II) biosorption onto WTC.

Δ*H*^o^ (kJ mol^−1^)	Δ*S*^o^ (J K^−1^ mol^−1^)	Δ*G*^o^ (kJ mol^−1^)		
		*T* = 20 °C	30 °C	50 °C
−8.9 ± 0.9	−22 ± 2.4	−2.5 ± 0.7	−2.3 ± 1.0	−1.8 ± 0.8

**Table 5:** Kinetic and isotherm models to evaluate Cd(II) biosorption onto WTC.

Kinetic Models	Equation	Parameters
Pseudo-first order	$q_{t}=q_{e}(1-e^{{-k}_{1}t})$	q_e_ (mg g^−1^): adsorption capacity q_t_ (mg g^−1^): the amount of Cd (II) retained per unit mass of biosorbent in time t. k_1_ (min^−1^): the first-order kinetic rate constant k_2_ (g mg^−1^ min^−1^): rate constant adsorption h: initial sorption rate (mg g^−1^ min^–1^)
Pseudo-second order	$q_{t}=\frac{{{q_{e}}^{2}k}_{2}\cdot t}{1+q_{e}\cdot k_{2}\cdot t}$ $h=k_{2}q_{e}^{2}$	
Intraparticle diffusion	$q_{t}=k_{id}t^{1/2}+C$	k_id_ (mg g^−1^ min^−1/2^): intraparticle diffusion rate constant C (mg g^−1^): constant related to the thickness of the adsorbent boundary layer
**Isotherm Models**	**Equation**	**Parameters**
Langmuir	$q_{e}=q_{max}\frac{K_{L}*C_{e}}{1+k_{L}C_{e}}$	C_e_ (mg L^−1^): adsorbate concentration in equilibrium q_max_ (mg g^−1^): Langmuir constant related to the maximum biosorption capacity K_L_: Langmuir constant related to the affinity between sorbent and sorbate
Freundlich	qe=KFCe1n	K_F_ (L^1/n^ mg^(n−1)/n^ g^−1^): constant equilibriumn n: constant related to the affinity between sorbent and sorbate.
Temkin	$q_{e}=Bl\;n(K_{T}C_{e})$	B = *RT*/b_T_, *R* is the gas constant (8.3145 J mol^−1^ K^−1^), *T* absolute temperature b_T_: Temkin constant related to the heat of adsorption (J mol^−1^) K_T_: Temkin isotherm equilibrium binding constant (L g^−1^)

## Data Availability

Not applicable.
